# Shifts in the Spatiotemporal Dynamics of Schistosomiasis: A Case Study in Anhui Province, China

**DOI:** 10.1371/journal.pntd.0003715

**Published:** 2015-04-16

**Authors:** Yi Hu, Rui Li, Yue Chen, Fenghua Gao, Qizhi Wang, Shiqing Zhang, Zhijie Zhang, Qingwu Jiang

**Affiliations:** 1 Department of Epidemiology, School of Public Health, Fudan University, Shanghai, China; 2 Key Laboratory of Public Health Safety, Ministry of Education, Shanghai, China; 3 Laboratory for Spatial Analysis and Modeling, School of Public Health, Fudan University, Shanghai, China; 4 School of Epidemiology, Public Health and Preventive Medicine, Faculty of Medicine, University of Ottawa, Ottawa, Canada; 5 Anhui Institute of Parasitic Diseases, Wuhu, China; 6 Biomedical Statistical Center, Fudan University, Shanghai, China; Case Western Reserve University School of Medicine, UNITED STATES

## Abstract

**Background:**

The Chinese national surveillance system showed that the risk of *Schistosoma japonicum* infection fluctuated temporally. This dynamical change might indicate periodicity of the disease, and its understanding could significantly improve targeted interventions to reduce the burden of schistosomiasis. The goal of this study was to investigate how the schistosomiasis risk varied temporally and spatially in recent years.

**Methodology/Principal Findings:**

Parasitological data were obtained through repeated cross-sectional surveys that were carried out during 1997-2010 in Anhui Province, East China. A multivariate autoregressive model, combined with principal oscillation pattern (POP) analysis, was used to evaluate the spatio-temporal variation of schistosomiasis risk. Results showed that the temporal changes of schistosomiasis risk in the study area could be decomposed into two sustained damped oscillatory modes with estimated period of approximately 2.5 years. The POPs associated with these oscillatory components showed that the pattern near the Yangtze River varied markedly and that the disease risk appeared to evolve in a Southwest/Northeast orientation. The POP coefficients showed decreasing tendency until 2001, then increasing during 2002-2005 and decaying afterwards.

**Conclusion:**

The POP analysis characterized the variations of schistosomiasis risk over space and time and demonstrated that the disease mainly varied temporally along the Yangtze River. The schistosomiasis risk declined periodically with a temporal fluctuation. Whether it resulted from previous national control strategies on schistosomiasis needs further investigations.

## Author Summary

We investigated changes in dynamics of schistosomiasis transmission over space and time in Anhui Province of East China. Parasitological data were obtained through repeated cross-sectional surveys that were carried out during 1997–2010. A multivariate autoregressive model, combined with principal oscillation pattern (POP) analysis, was used to evaluate the spatio-temporal variation of schistosomiasis risk. The schistosomiasis risk changed temporally as a damped oscillatory mode with a fluctuation, indicating that the disease risk declined periodically but with a temporal ascent during the study period. This change might result from national control strategies on schistosomiasis. The POP analysis also demonstrated a shifting spatial pattern of schistosomiasis along the Yangtze River. The POP method is commonly used in geosciences but not in epidemiology. Our analysis based on the approach provided new insights into the research of schistosomiasis transmission. The utility of such methods for addressing epidemiological problems will grow as more large-scale datasets become readily available.

## Introduction

Schistosoma infections remain a serious public health problem worldwide, infecting more than 200 million people in approximately 76 developing countries with a loss of 1.7 to 4.5 million disability-adjusted life years (DALYs) [1]. *Schistosoma japonicum*, one of the three main schistosome species, is responsible for human and animal infections in the People Republic of China. More than 50 million Chinese are currently at risk of infection [2]. Schistosomiasis remains endemic in many, limited foci in the hilly and mountainous regions in Sichuan and Yunnan provinces while major foci of endemicity occur in the lake and marshland areas (Poyang Lake and Dongting Lake) along the Yangtze River basin, where the elimination of transmission has proven difficult to achieve [3,4]. More than 80% of all current cases in the country are found in those areas [5].

Techniques of Geographic Information Systems (GIS) and Remote Sensing (RS) have been applied in a China’s national schistosomiasis control program over the past decades [6,7,8,9,10]. The Chinese government presents their annual reports on schistosome-endemic areas by means of digital maps, which helps the planning and operating of the national control program [11]. With the development of national and local surveillance systems, long-term temporal parasitological data are available, which provides us good opportunities to characterize the changing pattern of disease risk. The national surveillance system showed that the risk of schistosoma infection had decreased substantially by the end of the World Bank Loan Project (WBLP) in 2001 [12], rebounded shortly afterwards [13], declined again after 2005 and finally remained at a relatively low infection level currently [14]. This dynamic change might indicate periodicity of the disease risk within the intervention periods. Targeted interventions to reduce the burden of schistosomiasis could be significantly improved if periodicity of the disease is better understood. Unfortunately, there has been no evidence so far to support this assumption of periodicity in schistosomiasis transmission.

The goal of this study was to investigate how the schistosomiasis risk varied temporally and spatially in recent years and the paper is organized as follows. We first implement a multivariate autoregressive model to assess the changes in schistosoma infection in Anhui Province of China, using annual county-level prevalence data for the period 1997–2010. We employed the principal oscillation pattern (POP) analysis, a multivariate technique to empirically infer the characteristics of the space-time variations of a possibly complex system [15], to detect the spatio-temporal variation of schistosomiasis risk over our study period. Concluding remarks are finally presented with regards to the results.

## Materials and Methods

### Approach and study area

In the present study, we developed a vector autoregressive (VAR) model, combined with POP analysis, to evaluate the spatio-temporal variation in schistosomiasis incidence. The analysis was conducted at the county-level schistosomiasis data from Anhui Province. Anhui Province, located across the lower reaches of the Yangtze River in East China, spans approximately 139,600 square kilometers with a population of 60.83 million (2014). Plains dominate the province, with a series of hills and ranges covering southwestern and southeastern Anhui. Major rivers include the Huaihe River in the north and the Yangtze River in the south.

### Parasitological data

**Fig 1:**
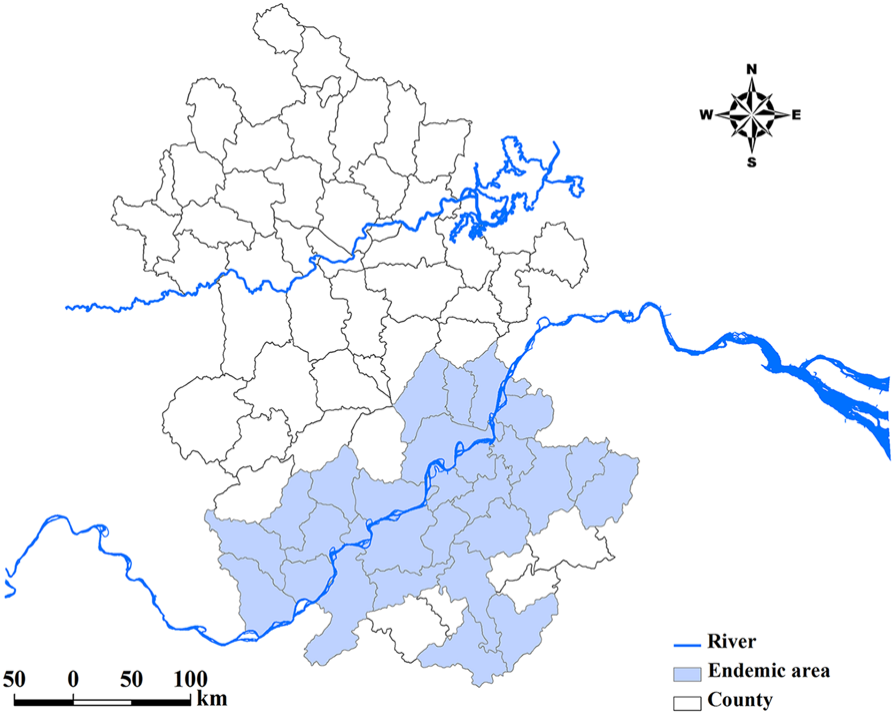
Endemic area of schistosomiasis japonica in Anhui Province, People’s Republic of China. The river in the north is the Huaihe River and the one in the south is the Yangtze River. The map was created using ArcGIS software (version 10.0, ESRI Inc. Redlands, CA).

The *S*. *japonicum* infection prevalence data were collected from repeated cross-sectional surveys carried out by the health professionals of the Anhui Institute of Parasitic Diseases annually between 1997 and 2010. These data were originally collected through village-based field surveys using a two-pronged diagnostic approach (all residents aged 5 to 65 years were screened by a serological test and then confirmed by a fecal parasitological test (Kato-Katz technique)) [16], with aggregated data available to us at the county level. For our spatio-temporal variation analysis, we removed the counties with zero prevalence of the disease during the study period and 31 schistosome-endemic counties were included in this study (Fig 1).

### Ethics statement

Approval for oral consent and other aspects of the surveys were granted by the Ethics Committee of Fudan University (ID: IRB#2011-03-0295). Written informed consent was also obtained from all participants.

### Statistical analysis

#### Multivariate autoregressive modeling

In order to model the dynamical pattern of schistosomiasis, we assume the data of yearly prevalence in each county follow a first-order VAR process (i.e., VAR(1)) and the state of the process at time *t* is described by a set of *n* variables, Z_t_ ≡ (Z_t_(1),…, Z_t_(*n*))′ (i.e., one variable corresponds to one county):

$$z_{t}=Mz_{t-1}+\varepsilon_{t}$$ (1)

where *M* ϵ ℜ^*N×N*^ denotes the transition matrix of evolving coefficients and *ε*_*t*_ ϵ ℜ^*N×N*^ denotes a temporally uncorrelated noise vector with mean 0 and covariance matrix Σ ϵ ℜ^*N×N*^. An *n*-variate AR(1) model can be cast in the form of a regression model and the parameters of M and *ε*_*t*_ estimated by least squares [17].

#### POP analysis

POP analysis yields dynamical modes from a spatio-temporal dataset through the analysis of a stochastic model fitted to the observations [18,19]. POP analysis assumes that the observed field (i.e., prevalence field in this study) has a temporal autoregressive structure of order one, as in formula (1), and involves a spectral decomposition of the transition matrix M. Consider the spectral decomposition:

$$M=WLV^{*}$$ (2)

where * denotes the Hermitian transpose (i.e., $V^{*}={\overset{-}{W}}^{-1}$, the inverse complex conjugate of *W*), W is comprised of the right singular vectors {*w*_1_, …, *w*_n_}, V* is comprised of the left singular vectors {v_1_, …, v_n_}, and L = *di ag*(*λ*_*k*_) is diagonal.

**Fig 2:**
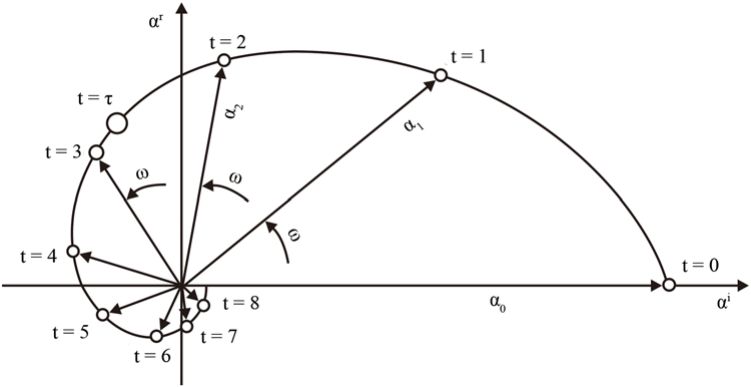
Schematic diagram of the time evolution of POP coefficients *α*_*t*_(*k*) with an initial value *α*_*0*_ = (*α* ^*r*^, *α* ^*i*^) = (0,1) (From [19]). The complex number *α*_*t*_ rotates in slightly more than eight time steps anticlockwise once around the origin. *ω* is a constant angle in the complex plane. The *e*-folding time *τ*, for which |*α*_*τ*_| = 1/*e* is marked by an open circle. The amplitude |*α*_*t*_|, in the perspective of epidemiology, corresponds to magnitude of schistosomiasis risk in our study.

The eigen-decomposition of M yields the dominant modes of variability from the multivariate dataset in terms of relaxation and oscillation modes. The vectors *w*_k_ are called the principal oscillation patterns or system normal modes, while the left singular vector *v*_k_ (the columns of matrix V) are called adjoint bases. The elements of the time series *α*_*t*_ = V*Z_*t*_ are known as POP coefficients. In the spatio-temporal setting, *w*_k_ provides a spatial map of variability of the observed field. If the eigenvalue *λ*_*k*_ (the element of diagonal matrix L) is complex, then $\lambda_{k}\equiv \lambda_{k}^{R}+i\lambda_{k}^{I}$ (where $i\equiv \sqrt{-1}$ and *k* = 1,…,*m*), which can be written in polar form as $\lambda_{k}^{R}=\gamma_{k}cos(\omega_{k})$ and $\lambda_{k}^{I}=\gamma_{k}sin(\omega_{k})$. Then, *λ*_*k*_ = *γ*_*k*_(COS(*ω*_*k*_)+*i* sin(*ω*_*k*_)), where $\gamma_{k}=|\lambda_{k}|={\left\{{\left(\lambda_{k}^{R}\right)}^{2}+{\left(\lambda_{k}^{I}\right)}^{2}\right\}}^{1/2}$ and $\omega_{k}=arctan(\lambda_{k}^{I}/\lambda_{k}^{R})$. Thus, the POP coefficients evolve according to,$\alpha_{t}(k)=\gamma_{k}^{t}(cos(\omega_{k}t)+isin(\omega_{k}t))$. Under the condition, *γ*_*k*_≤1; *α*_*t*_ evolves as a damped spiral in the complex plane (Fig 2) with a characteristic damping rate *γ*_*k*_ and frequency *ω*_*k*_, for *k* = 1,…,*m*. Notice that *ω*_*k*_ here is an angle in the complex plane. In the case that *λ*_*k*_ is real with module less than 1, *α*_*t*_ corresponds to damped non-oscillatory modes. In both cases, the amplitude of *α*_*t*_(*k*) (i.e.,$\left|\lambda_{k}^{t}\right|$) decreases exponentially with time *t* and can be characterized by the “*e*-folding time” (the time needed to reduce the initial amplitude *α*_*0*_(*k*) to *α*_*0*_(*k*)/*e*), *τ*_*k*_ = -1/log(*γ*_*k*_).

We decompose the right singular vector into its real and imaginary parts as:$w_{k}=w_{k}^{R}+iw_{k}^{I}$. Note that for M real, the normal modes occur in complex conjugate pairs if they are complex. Hence, the general evolution of a damped normal mode (i.e., *γ*_*k*_<1) can be described in a two-dimensional subspace spanned by $w_{k}^{R}$ and $w_{k}^{I}$; it occurs in the succession,

$$..\to w_{k}^{R}\to -w_{k}^{I}\to -w_{k}^{R}\to w_{k}^{I}\to w_{k}^{R}\to ...$$ (3)

with a period of 2*π*/*ω*_*k*_; and each stage in (3) occurs a quarter of a cycle apart.

#### Data preprocessing

Difficulties in the estimation of an *n*-dimensional AR(1) are often associated with the large number of parameters involved in formula (1). For large fields, the number of spatial degrees of freedom (i.e., n-dimensional vector z_t_) can be reduced by considering the leading components from a principal component analysis (PCA) which is a useful multivariate analysis method for extraction of the dominant variability patterns from an observed field [20], thereby reducing dimensionality while retaining most of the variance in the original field. A positive by-product of fitting a multivariate autoregressive model in PCA space is the exclusion of noisy components from the analysis and diagonalization of the error covariance matrix. Before PCA was performed, we standardized the prevalence data (i.e., subtracted the mean and divided by the standard deviation) to reduce possible domination by raw prevalence with larger variances.

## Results

**Fig 3:**
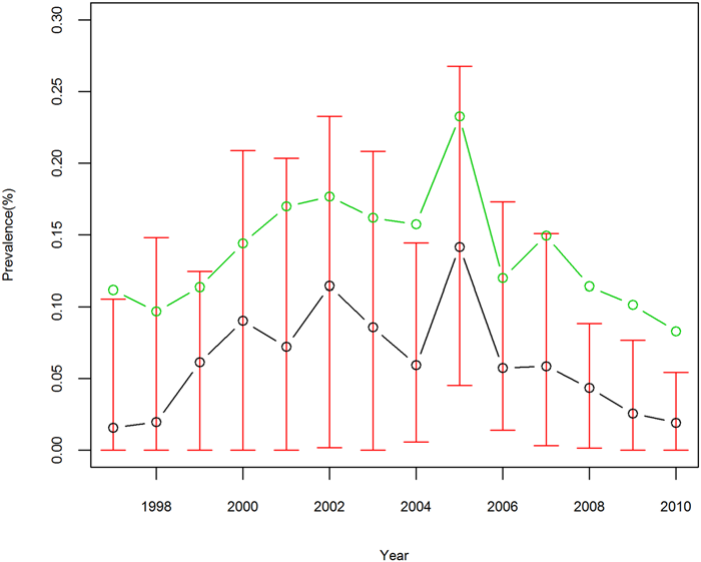
Prevalence of *S*. *japonicum* infection for counties in Anhui Province, China, from 1997 to 2010. The red vertical lines denote interquartile range, the green circles denote the mean, and the black circles denote the median.

Fig 3 provides the annual prevalence of schistosomiasis during the study period. The mean prevalence gradually increased from 0.11% in 1997 to 0.23% in 2005, and then decreased substantially to 0.08% in 2010. This fluctuated tendency was accompanied by a corresponding variation in prevalence across counties with the interquartile range (IQR) expanding from 0–0.02/100 in 1997 to 0.04–0.28/100 in 2005 and then shrinking to 0–0.05/100 in 2010. A similar trend was observed when median prevalence was used.

**Fig 4:**
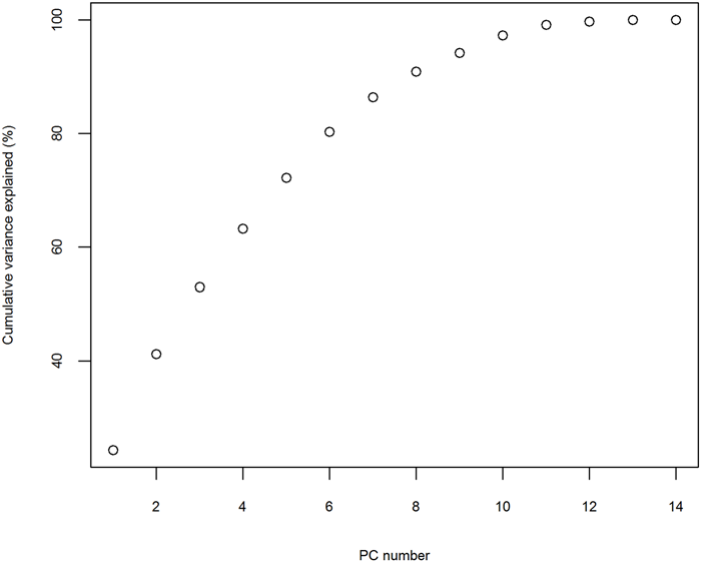
Fraction of variance accounted by the principal components (PC).

As noted above, the large number of spatial degrees of freedom can be reduced by considering a truncated PCA version of the original prevalence field. PCA shows that a subspace of 7 principal components (PCs) explain 86.40% of the overall prevalence field variation (Fig 4), with the first and the second PCs accounting for 24.27% and 16.94% respectively. The estimation of the parameters in formula (1) is, therefore, carried out in the PAC subspace by least squares.

**Fig 5:**
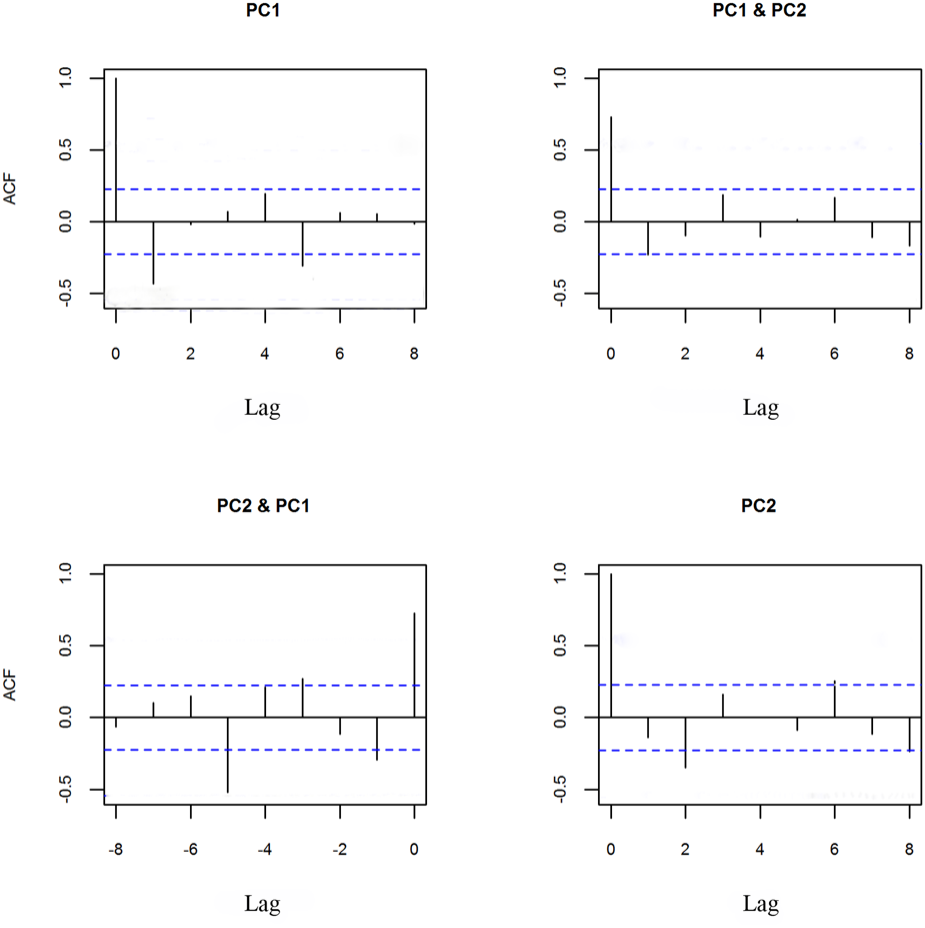
Correlation of residuals from the formula (1). Top left: autocorrelation for PC1; Top right: cross-correlation between PC1 and PC2 (positive lag); Bottom right: cross-correlation between PC1 and PC2 (negative lag); Bottom right: autocorrelation for PC2. Horizontal dashed lines represent 95% confidence levels for correlation of white noise realizations. Lags are spaced by one year.

Uncorrelatedness of the residuals in formula (1) is a primary criterion for checking the adequacy of an estimated model. The autocorrelation function (ACF) for residuals of the first (the top left in Fig 5) and second PCs (the bottom right in Fig 5) indicates that the model yields an uncorrelated residual series. The rest of Fig 5 show that the estimated model is able to describe the relationship between the first and second components of the observed field yielding uncorrelated residual series. Similar analyses were carried out for the rest of PCs and uncorrelated residuals were found.

We assessed the dynamics of the prevalence field by examining the POPs of the matrix M. Table 1 summarizes the eigenvalues from the POP decomposition. All the modules of eigenvalues are less than one, indicating a damped mode for the whole system, with six PCs (corresponding to six complex eigenvalues) showing an oscillatory mode and one PC (corresponding to one real eigenvalue) showing a non-oscillatory mode. The oscillation frequencies varied moderately, with estimated periods in approximately 2 and 14 years and the corresponding *e*-folding time decreased from 4.71 to 1.01 years. Only sustained modes (i.e., POP1 and POP2), as measured by the corresponding *e*-folding time (*e*-folding > a quarter of period) [21], were considered in the further POP analysis.

**Table 1:** Eigenvalues from matrix M in formula (1).

POPs*	Eigenvalue	Period (years)	*e*-folding time (years)
1st	-0.74+0.33i	2.31	4.71
2nd	-0.74–0.33i	2.31	4.71
3rd	0.66+0.32i	13.95	3.15
4th	0.66–0.32i	13.95	3.15
5th	0.51	_	1.50
6th	0.20+0.31i	6.28	1.01
7th	0.20–0.31i	6.28	1.01

*POPs: principal oscillatory patterns.

**Fig 6:**
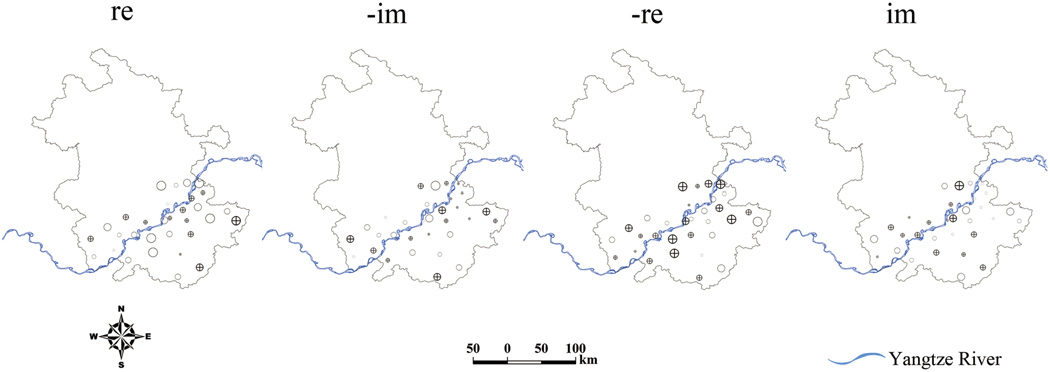
The first normal modes plotted spatially for the matrix M. As POP2 and POP1 are conjugate vectors, only POP1 is shown for the pair. Open circles denote negative values, while circles with pluses represent positive values; larger circles imply larger magnitude and small circles are values close to zero. The center points of the circles are located on centroids of county polygons. The shift of the spatial pattern of the disease within one period could be described from left to right. The maps were created using ArcGIS software (version 10.0, ESRI Inc. Redlands, CA).

**Fig 7:**
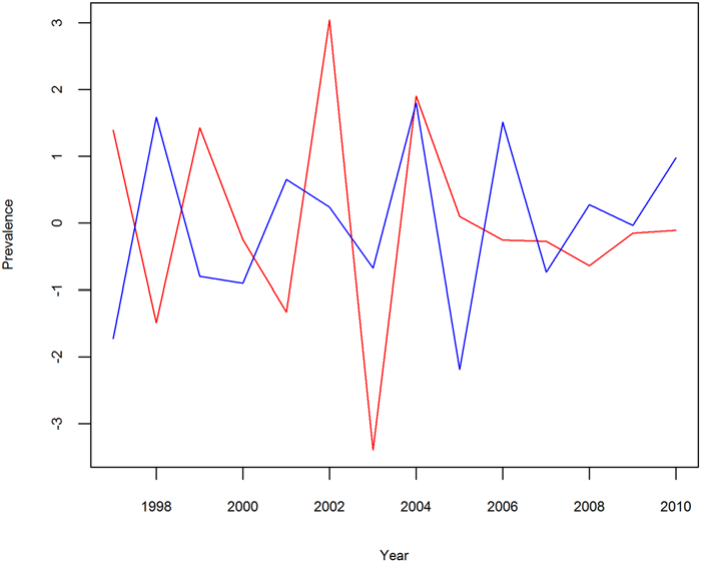
POP coefficients at(k). As a_t_(1) and a_t_(2) are conjugate, only one is shown for the pair. Plot A corresponds to POP1. The real part of the POP coefficient is shown in blue and the imaginary part is shown in red. The amplitude, in the perspective of epidemiology, corresponds to magnitude of schistosomiasis risk in our study. The y axis shows amplitude in units of 0.1%, and the x axis shows time in units of year.

The POPs associated with oscillatory components are displayed in Fig 6. It illustrates the evolving spatial patterns of POP1 as indicated in (3). The POP showed a similar trend that there was a large variation in areas close to the Yangtze River. An interesting feature is that the pattern of the disease risk appeared to evolve in a Southwest/Northeast orientation. Fig 7 shows the POP coefficients (a_t_(k)) associated with POPs in Fig 6. The plot, as a whole, shows a damped but fluctuated trend. The time series associated with POP1 showed decreasing amplitude until 2001, then increasing amplitude during 2002–2005 and decaying amplitude afterwards. The time series showed short and decaying amplitude at the end of the study period.

## Discussion

This study presents an application of POP analysis to detect and characterize the changes in dynamics of schistosomiasis transmission over space and time in Anhui Province during 1997–2010. POP analysis is a useful method for assessing the structure of the VAR dynamical systems, which has been extensively used in geosciences to empirically infer the characteristics of the space-time variations of a complex physical system [18]. Our analysis was motivated by the perspective of evolution of the physical (disease) process over space and time. Our results showed that the schistosomiasis risk varied with a damped and oscillatory mode, and there was theoretically a trend towards eradication though the true eradication, depending on a combination of dynamics, control, and public health response to the current situation, is still a long way away. Previous studies [22,23,24,25] have investigated the spatio-temporal variation of schistosomiasis or the spatial pattern of disease that varied yearly, but seldom have summarized the change. This is the first study, to our knowledge, that attempts to address this issue.

The damped oscillation in physics refers to the motion state of an oscillatory system (i.e., a physical system in which repetitive variation occur between the state of equilibrium) that suffers some sort of irreversible energy loss while it is in motion due, for instance, to kinetic friction and become damped. The energy conserved in the whole system can be reflected by the amplitude of the oscillation system. In our study, this mode was found in the prevalence-field system (an analogy of physical system), which suggested that the variation of the schistosomiasis risk presented a periodic change and “lost energy” during the transmission due to various factors, and therefore the disease risk declined, which is reflected by the amplitude of POP coefficients (i.e., a_t_(k)). From the practical perspective, we can name the damped oscillation in physics as "periodically declined mode".

Of the seven modes of prevalence variation, we focused on the sustained modes (i.e., POP1 and POP2) as measured by the corresponding *e*-folding time (*e*-folding > a quarter of period) when interpreting the space-time dynamics of schistosomiasis. The *e*-folding time is the time required for the amplitude of a damped oscillation to decay to 1/e of its initial value. The smaller the *e*-folding time the more damped is the oscillation. If the *e*-folding time is too short (and shorter than a quarter of the period), the oscillation “fades away” too fast before completing the first one-period cycle. For example, the *α*_*t*_(3), corresponding to POP3, cannot complete its first period of 14 years as the risk (reflected by amplitude) become nearly vanished as only 1/*e* ^3^ (i.e., about 5%) of the initial risk remains after three *e*-folding times (i.e., 9.45 years). Therefore, we are only interested in such relatively “permanent” oscillations as POP1 and POP2.

The first and second eigenvalues of the transition matrix M (Table 1), associated with POP1 and POP2 respectively, showed that over 40% (Fig 4) of the prevalence variation in 1997–2010 could be described by a periodically declined mode with a period of 2.31 years. Combined with the plots in Fig 7, this mode indicated that the schistosomiasis risk, explained by POP1 and POP2, presented a declining tendency of almost 2.5-year period with a fluctuation. The Southwest/Northeast oriented shift of disease’s spatial pattern, explained by the leading two POPs within one period, indicated that the disease varied temporally along the Yangtze River (Fig 6). The possible explanation for this change would be the frequent and uncontrolled flooding caused by the Yangtze River, which continued to be a prime risk factor for schistosomiasis [26,27]. With regular flooding, snails in these habitats could be dispersed and subsequently deposited widely in various localities, such as lake and wetland, and individuals came into contact with such contaminated localities, leading to more infections with schistosome.

The declined but fluctuated plots of POP coefficients (a_t_(k)) in Fig 7 gave insights into the temporal variation of schistosomiasis risk. The imaginary and real part of the time series in the plot show increased amplitude successively during 2002–2005, reflecting a greater risk compared to the rest of the study period. There were two important national control strategies implemented in the history of fighting against schistosomiasis in China. A 10-year World Bank Loan Project (WBLP) was launched in 1992 to boost schistosomiasis control [28], which employed large-scale chemotherapy. By the end of the WBLP in 2001, the prevalence of the disease was greatly reduced [13], which is reflected by decreasing amplitude during 1997–2001. However, the disease rebounded shortly after the conclusion of the WBLP [3,29], which corresponded to the abruptly increased amplitude since 2002. This abruptly increased amplitude change further implies that the burden schistosomiasis risk was even heavier than before. In response to the rebound of the disease, a national control program with a revised strategy to control schistosomiasis using integrated measures with the source of infection control as an emphasis had been implemented since 2005 [14]. The program included such strategies as replacement of plowing cattle with machines, treatment of night-soil and safety water supply, breeding of domestic animals in barns, change of snail habitats with the construction of water conservancy projects, among others [30,31]. Between the two national programs, each endemic region was only based on the available funding to decide their respective control strategy [32]. The short and decaying amplitude since 2006 indicates a decreased disease risk and this might suggest the latter program is effective and should continue.

An assumption has been made in our analysis that the dynamical attribute of the prevalence field can be modeled using a VAR(1) process. Although this VAR process is generally considered to be linear, non-linear dynamics can be built in a general case where the transition matrix M is time-varying (i.e., M_t_). However, the model validation (i.e., ACF) indicates that the linear model (1) fits the data well. Only two sustained modes (i.e., POP1 and POP2) have been inferred from the VAR(1) modeling, which is a limitation of our dataset, i.e., strongly constrained by the short length (14 years) of the schistosomiasis prevalence series. Another limitation is that we were not able to directly line the spatio-temporal changes in schistosomiasis prevalence to the two national control strategies and other factors (e.g., climate change and changing water level) in the current analysis. Further studies are needed to understand the observed periodicity in the schistosomiasis transmission and declining disease risk with a fluctuation over time.

The characterization of dynamical shifts in annual transmission of schistosomiasis can be epidemiologically important from a control perspective. In order to thoroughly assess such shifts in dynamics in a given region, spatially explicit parasitological data should be considered. Here, we have adopted a VAR model as well as a model-output analysis method (i.e., POP) that allows us to determine if shifts in the system (i.e., prevalence field) dynamics exist and to characterize them. Our analysis illustrated how schistosomiasis risk varied across space and time and demonstrated that annual variation of the disease during the study period could be decomposed into several periodical modes and this could be useful in forecasting transmission at various locations although the focus here is assessing the changes in dynamics of schistosomiasis risk. The Southwest/Northeast orientation. Policy makers may find the spatio-temporal variation of schistosomiasis under the implementation of a control strategy useful for managing the disease.

The POP analysis, although new in the field of epidemiology, is popular in fields with obvious dynamical systems (e.g., fluid dynamics in geosciences). High-dimensional spatio-temporal research is less common in epidemiology due mostly to the lack of availability of corresponding epidemiological data. However, as more local and national surveillance systems are established, there would be more large-scale datasets available for this type of analysis in various disciplines. We believe our study provides a good example for this direction.
